# Adipogenesis-Related Metabolic Condition Affects Shear-Stressed Endothelial Cells Activity Responding to Titanium

**DOI:** 10.3390/jfb14030162

**Published:** 2023-03-17

**Authors:** Thaís Silva Pinto, Anderson Moreira Gomes, Paula Bertin de Morais, Willian F. Zambuzzi

**Affiliations:** Lab. of Bioassays and Cellular Dynamics, Department of Chemical and Biological Sciences, Institute of Biosciences, UNESP—São Paulo State University, Botucatu 18618-970, SP, Brazil

**Keywords:** bone, wound healing, adipogenesis, obese, dental implants, titanium, failure, angiogenesis

## Abstract

Purpose: Obesity has increased around the world. Obese individuals need to be better assisted, with special attention given to dental and medical specialties. Among obesity-related complications, the osseointegration of dental implants has raised concerns. This mechanism depends on healthy angiogenesis surrounding the implanted devices. As an experimental analysis able to mimic this issue is currently lacking, we address this issue by proposing an in vitro high-adipogenesis model using differentiated adipocytes to further investigate their endocrine and synergic effect in endothelial cells responding to titanium. Materials and methods: Firstly, adipocytes (3T3-L1 cell line) were differentiated under two experimental conditions: Ctrl (normal glucose concentration) and High-Glucose Medium (50 mM of glucose), which was validated using Oil Red O Staining and inflammatory markers gene expression by qPCR. Further, the adipocyte-conditioned medium was enriched by two types of titanium-related surfaces: Dual Acid-Etching (DAE) and Nano-Hydroxyapatite blasted surfaces (nHA) for up to 24 h. Finally, the endothelial cells (ECs) were exposed in those conditioned media under shear stress mimicking blood flow. Important genes related to angiogenesis were then evaluated by using RT-qPCR and Western blot. Results: Firstly, the high-adipogenicity model using 3T3-L1 adipocytes was validated presenting an increase in the oxidative stress markers, concomitantly with an increase in intracellular fat droplets, pro-inflammatory-related gene expressions, and also the ECM remodeling, as well as modulating mitogen-activated protein kinases (MAPKs). Additionally, Src was evaluated by Western blot, and its modulation can be related to EC survival signaling. Conclusion: Our study provides an experimental model of high adipogenesis in vitro by establishing a pro-inflammatory environment and intracellular fat droplets. Additionally, the efficacy of this model to evaluate the EC response to titanium-enriched mediums under adipogenicity-related metabolic conditions was analyzed, revealing significant interference with EC performance. Altogether, these data gather valuable findings on understanding the reasons for the higher percentage of implant failures in obese individuals.

## 1. Introduction

Physiological changes in obese patients are widely studied due to their systemic complications. The comorbidities known to be associated with obesity include cardiovascular, pulmonary, renal, endocrine, and musculoskeletal problems (e.g., arthrosis, osteoarthritis, back and joint pain), as well as impaired wound healing [1]. It is known that the adipose tissue develops endocrine activity by secreting bioactive substances, named adipokines, that can reach all systems and develop important roles in metabolism [2]. In obese individuals there is a dysfunction of the adipose tissue, presenting higher adipogenesis (high number and size of adipocytes, as well as intracellular fat droplets) that directly affects the profiles of released adipokines, including an increase in proinflammatory interleukin secretion, which triggers different responses systemically, including in the cardiovascular system and vasculature [3].

The vasculature exerts an important function in supplying blood to the whole body, delivering oxygen, nutrients, and essential factors to ensure adequate physiologic and metabolic functioning, also guaranteeing adequate tissue regeneration and repair in cases of injuries, when the vasculature also provides undifferentiated cells [4]. In this context, the endothelial cells (ECs), present along the luminal surface of the blood vessels, develop a central role in vascular activity by perceiving and responding to the changes in chemical and mechanical factors in the blood [5,6,7,8]. It is important to mention that angiogenesis is a decisive event during tissue healing [9] and it has been shown that ECs differently respond to metallic implantable medical devices, such as titanium alloys [10,11,12].

Titanium is commonly used as a biomaterial in several dental and medical fields, e.g., implantology, orthopedics, cardiology, and gastroenterology [13]. This metallic alloy is considered the gold standard for these purposes because it possesses important properties such as mechanical and corrosion resistance and proper biocompatibility [14]. After biomaterial implantation, the reactional tissue surrounding the implants suffers adaptation processes that require blood supplements through the blood vessels, highlighting the importance of EC activities and angiogenesis [15]. In addition, studies have revealed that angiogenesis and osteogenesis are coupled processes, and shown that better osseointegration occurs by interfering with the process of appositional new bone growth [16]. This coupling between cells seems to be affected in specific metabolic conditions, such as diabetes and obesity.

In fact, obesity is related to severe complications in the process of bone healing and osseointegration, likely because it provokes a burst of pro-inflammatory involvement, and an increased risk of bone loss [17]. The explanation for the obesity-related tissue regeneration complications lies in the effects of the unbalanced adipose-derived proinflammatory cytokines and adipokines [18], however, the biology involved in this context is barely known.

Thus, studies exploring the metabolic effects of high adipogenicity on different systems are needed, as well as strategies and alternative technologies and methodologies to study these conditions and predict biological responses. In this context, in vitro methods and analyses, such as cell culture, have the relevant advantages, despite presenting a huge set of limitations in comparison to in vivo protocols arising from age, genetics, environments, habits, hormones, etc. Furthermore, many in vitro methodologies capable of mimicking or closely addressing scenarios in biological systems, as well as in the presence of pathologies, have been proposed and used to guide preclinical experiments.

Using in vitro methodologies, we investigated the potential crosstalk between adipogenicity-related metabolic conditions and ECs responding to a titanium-enriched medium. Specifically, we demonstrate the proof of concept of the creation of the high-adipogenicity model using an adipocyte cell line, and observed its interference with EC activity. Altogether, these data gather new findings to understand the higher rate of failure of implants in obese individuals.

## 2. Methods

### 2.1. Implants

This experimental workflow was performed using two titanium surfaces, as follows: Dual Acid-Etched (DAE) and nanohydroxyapatite-coated surfaces (nHA). Both set of titanium discs were kindly provided by S.I.N.—Sistema Nacional de Implantes (Sao Paulo, SP, Brazil). The nHA titanium surface is described in more detail elsewhere by Gottlander et al. (1997) and Meirelles et al. (2008) [19,20].

### 2.2. Reagents

Dulbecco’s modified Eagle’s medium (DMEM), Fetal Bovine Serum (FBS), trypsin, penicillin, and streptomycin (antibiotics) were obtained from Nutricell (Campinas, Sao Paulo, Brazil). Trypan Blue (T6146), acetic acid glacial (695092), (3-(4,5-dimethylthiazol-2-yl)-2,5-diphenyltetrazolium bromide) (MTT) (M2128), ethanol (459844), Oil Red O (O0625), 3-isobutyl-1-methylxanthine (IBMX) (I5879), Dexamethasone (D1756), β-glycerophosphate (G9422), glucose (G-5400) and insulin (I2643) were obtained from Sigma Chemical Co. (St. Louis, MO, USA). The antibodies #9102, #8690, #3700, and #2109 were obtained from Cell Signaling Technology (Beverly, MA, USA). TRIzol™ reagent (15596026), DNase I (18068015), and the High-Capacity cDNA Reverse Transcription Kit (4368814) were purchased from Thermo Fisher Scientific Inc. (Waltham, MA, USA). GoTaq qPCR Master Mix (A6002) was obtained from PROMEGA (Madison, WI, USA). Oligonucleotides for gene expression were purchased from Exxtend (Campinas, São Paulo, Brazil).

### 2.3. Cell Culture

Two cell lines were used in this study: both 3T3-L1 preadipocytes (passage < 10) and Human Umbilical Vein Endothelial Cells (HUVECs; ECs) (ATCC; CRL1730; passage < 10) were cultured in Dulbecco’s modified Eagle’s medium (DMEM—Nutricell, Campinas, Brazil) containing penicillin 100 U/mL, streptomycin 100 mg/mL, and 10% fetal bovine serum (FBS). The adipocyte differentiation is described below. Importantly, ECs were cultured subjected to a shear stress model, as is described in detail below. In all experiments, the cells were maintained at 37 °C with a 5% CO_2_ and 95% humidity environment.

### 2.4. Adipocyte Differentiation

The 3T3-L1 adipocyte cultures were maintained in 100 mm culture dishes in DMEM supplemented with 10% FBS and 1% penicillin/streptomycin at 37 °C, 5% CO_2_, and 95% humidity until reaching proper confluence. Then, adipocyte differentiation was induced by supplementing the cell culture medium with insulin (1 mg/mL), dexamethasone (1 mM), and 3-isobutyl-1-methylxanthine (IBMX) (0.5 mM), which was used to expose the cells for 3 days. The medium was changed to maintenance DMEM containing 10% FBS and insulin (1 mg/mL) for an additional 7 days (completing 10 days in toto), being changed every 2 days in the meantime. This differentiation process was followed under 2 separate experimental conditions; one under normal conditions (Ctrl), and the other subjected to a high-glucose medium (HGM) to induce higher adipogenesis, reaching a final glucose concentration of 50 mM. The cultures were maintained up to 10 days, at which point the cells were harvested and the conditioned medium collected to further expose ECs.

### 2.5. Shear Stress Model

**Figure 1:**
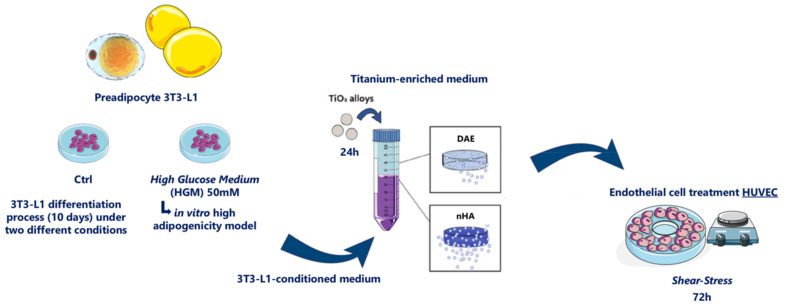
Outline and workflow. To evaluate the ECs behavior in response to titanium-enriched medium in concomitance with high adipogenesis conditions arising from 3T3-L1 adipocytes, we collected the medium conditioned by the adipocytes during their differentiation, which was later enriched with titanium for up to 24 h, as recommended by ISO 10993:2016. This conditioned medium was also further used to expose ECs for 72 h under shear stress mimicking blood flow, at which point the samples were collected to allow the molecular analysis.

ECs were seeded in the peripheral area of previously modified 100 mm culture dishes (Figure 1). The modification in the 100 mm culture dishes was made by using medical silicone to bond a 60 mm culture dish at the center-bottom of the 100 mm culture dishes. Thereafter, the modified dishes were sterilized using UV light for 30 min. To perform the model, the ECs were exposed to the tension forces induced by the circuit of shear stress triggered by the rotations of an orbital shaker (Scilogex, Rocky Hill, CT, USA) placed in the cell culture incubator, as in other experiments [21,22]. We calculated the maximal wall shear stress of ~3 Pa (physiological arterial shear stress = ~1–4 Pa) by using this equation: τmax = α√ρη(2πʄ)3. This equation encompasses the τmax that is the shear stress (Pascal), α is the radius of orbital rotation (12 cm), ρ is the density of the cell culture medium (937.5 kg/m^3^), η is the viscosity of the cell culture medium (7.5 × 10^−4^ Pa s), and ʄ is the frequency of rotation.

### 2.6. Cell Viability Assay

The high-glucose medium was prepared previously at 50 mM final concentration. Concomitantly, the 3T3-L1 adipocytes were maintained on 96-well plates (5 × 10^4^ cells/mL) and later incubated for up to 24 h. One group of cells was treated with the HGM to evaluate whether the high glucose concentration would affect cell viability. The control group contained cells under normal cell culture conditions. The cells were maintained in both treatments (Ctrl and HGM 50 mM) for up to 72 h, after which the cell viability was measured by adding 1 mg/mL of 3-(4,5-dimethyl-2-thiazolyl)-2,5-diphenyl-2H-tetrazolium bromide (MTT) to evaluate the mitochondrial dehydrogenase activity through the MTT reduction reaction after 3 h in a CO_2_ incubator. During this reaction, the yellow-colored water-soluble tetrazolium salt MTT becomes the purple-colored soluble compound formazan proportional to mitochondrial activity. The dye of formazan was later dissolved in DMSO, and the absorbance was measured at 570 nm (Synergy II; BioTek Instruments, Winooski, VT, USA).

### 2.7. Oil Red O Staining

Previously, Oil Red O solution was obtained by dissolving the Oil Red O dye in propylene glycol (0.5%; *w*/*v*) in a heater at 95 °C. By using a 0.45 μm syringe filter the Oil Red O solution was filtered to eliminate residual particulates from the solution and later used to stain the adipocytes. Adipocytes were differentiated for 10 days using a 24-well plate. At the end of differentiation, the medium was removed, the cells were washed with warm PBS, fixed in 4% paraformaldehyde for 10 min at room temperature, washed twice in deionized water, and then maintained in absolute propylene glycol for 5 min. The cells were stained in Oil Red O solution 0.5% up to 30 min at room temperature, then washed in 85% propylene glycol solution for 3 min. Finally, 3T3-L1 was washed twice in deionized water. Images were acquired using an inverted microscope (Axio Vert.A1, Carl Zeiss microscopy GMBH, Göttingen, Germany).

### 2.8. Titanium-Enriched Medium Obtaining

Adipocyte-related conditioned medium was later used to incubate both types of titanium disc for 24 h: DAE and nHA, in accordance with ISO 10993:2016 (0.2 g/mL *w*/*v*) with slight modification as suggested by Zambuzzi et al. [23,24,25,26]. The final conditioned medium was later used to expose ECs for 3 days under shear stress to mimic blood flow.

### 2.9. Western Blot

Both adipocytes and ECs were harvested using lysis buffer [Lysis Cocktail (50 mM Tris [tris(hydroxymethyl)aminomethane]–HCl [pH 7.4], 1% Tween 20, 0.25% sodium deoxycholate, 150 mM NaCl, 1 mM EGTA (ethylene glycol tetraacetic acid), 1 mM *O*-Vanadate, 1 mM NaF, and protease inhibitors [1 μg/mL aprotinin, 10 μg/mL leupeptin, and 1 mM 4-(2-amino-ethyl)-benzolsulfonylfluorid-hydrochloride])] for 2 h, after which the samples were cleared by centrifugation, and the protein concentration was measured using the Lowry method [27]. An equal volume of 2x sodium dodecyl sulfate (SDS) gel loading buffer (100 mM Tris-HCl [pH 6.8], 200 mM dithiothreitol [DTT], 4% SDS, 0.1% bromophenol blue, and 20% glycerol) was added to the samples and boiled for 5 min. Aliquots of protein extracts were resolved into SDS-PAGE (10 or 12%) and transferred to PVDF membranes (Millipore, Burlington, MA, USA). Membranes were blocked with either 5% fat-free dried milk dissolved in Tris-buffered saline (TBS)–Tween 20 (0.05%) and incubated overnight at 4° C with appropriate primary antibody at 1:1000 dilutions. After washing 3x TBS-Tween 20 (0.05%), those membranes were incubated with horseradish peroxidase-conjugated secondary IgGs antibodies, at 1:5000 dilutions, in a blocking buffer for 1 h. Thereafter, the bands were detected by enhanced chemiluminescence (ECL), or by fluorescence (ODYSSEY^®^ CLx Infrared Imaging System).

### 2.10. Quantitative PCR Assay (qPCR)

The same experimental workflow was performed and the cells were harvested now in Ambion TRIzol Reagent (Life Sciences—Fisher Scientific Inc, Waltham, MA, USA), and treated with DNase I (Invitrogen, Carlsband, CA, USA). cDNA synthesis was performed using High-Capacity cDNA Reverse Transcription Kit (Applied Biosystems, Foster City, CA, USA) following the manufacturer’s instructions. qPCR was performed on a total of 10 μL, containing PowerUp™ SYBR™ Green Master Mix 2x (5 μL) (Applied Biosystems, Foster City, CA, USA), 0.4 μM of each primer, and 50 ng of cDNA and nuclease-free H_2_O. Data were expressed as relative amounts of each target gene normalized considering the expression of 18SrRNA and Gapdh genes, here used as housekeeping genes, using the cycle threshold (Ct) method. Specific primers and running details are described in Table 1.

### 2.11. Oxidative Stress Markers

After obtaining adipocytes, the cells were harvested in PBS and sonicated. Protein carbonylation (CBO) was measured by using DNPH (2,4-dinitrophenyl hydrazine) as derivatizing agent [28]. The experiment was performed in a dark chamber to prevent the light. Firstly, the samples (10 µL of the lysed cells) were incubated with DNPH 10 mM (100 µL) for 10 min, after which 50 µL of 6 M NaOH (sodium hydroxide) was added. The reaction was interrupted after 10 min. The CBO was estimated by reading the final solution coloration spectrophotometrically at 450 nm. Finally, the results were calculated using the molar extinction coefficient (22,000 M^−1^ cm^−1^) of DNPH and expressed as nmol/mg protein.

### 2.12. Matrix Metalloproteinases (MMPs) Activities by Zymography

Differentiating adipocyte cell culture medium was collected to measure the activity of matrix metalloproteinases (MMPs). The conditioned medium was centrifuged at 14,000 rpm for 15 min to avoid cell debris, and the protein concentration was determined using the Lowry method [27]. The same concentration of protein was resolved into a 12% polyacrylamide gel containing 4% gelatin. The gelatinolytic activity of MMPs was determined in the resolved proteins (bands). The proteins’ structures were renatured in Triton X-100 aqueous solution (2% *w*/*v*) for 40 min, followed by incubation for 18 h in proteolysis buffer (Tris-CaCl_2_) at 37 °C, when the gels were stained using Coomassie Blue R-250 dye solution 0.05% for 3 h. Thereafter, the stained gels were washed in a 30% methanol (*v/v*) and 10% glacial acetic acid solution (*v*/*v*). The opposite staining (clear bands) was obtained in the gels exactly where there was the gelatinolytic activity (bands) of MMP2 (~62 kDa) and MMP9 (~84 kDa), and then they were analyzed using the software ImageJ (Bethesda, MD, USA), as previously proposed [29].

### 2.13. Statistical Analysis

Data were expressed as mean ± standard error of the mean (SEM) of the replicates of each experiment (n = 3). The samples assumed a normal distribution, and they were subjected to Student’s *t*-test (two-tailed) with *p* < 0.05 considered statistically significant. In the experiment where there were more than two groups, the statistical analyses were performed using either analysis of variance (one-way ANOVA) combined with appropriate Bonferroni’s correction post-test, or nonparametric analysis. A *p* < 0.05 was considered to be statistically significant. The software used was GraphPad Prism 7 (GraphPad Software, La Jolla, CA, USA).

## 3. Results

To better evaluate the molecular mechanism underlying EC response to high-adipogenesis conditions concomitant to a titanium-enriched medium, we proposed an in vitro experimental model subjecting adipocyte cells to differentiation under two conditions: Control cultures (Ctrl) and high-glucose treated cells (H_Adip). Furthermore, the adipocyte-obtained medium was enriched by titanium and then used to expose semiconfluent EC cultures dynamically responding to mechanotransduction mimicking blood flow [22]. Additionally, the titanium-enriched medium was obtained using two different titanium-modified surfaces: DAE, in which the discs were subjected to dual acid-etching, and nHA, in which DAE surfaces were covered by nano-hydroxyapatite [11,30,31], as we have shown previously.

### 3.1. Validation of the High-Adipogenesis Model

**Figure 2:**
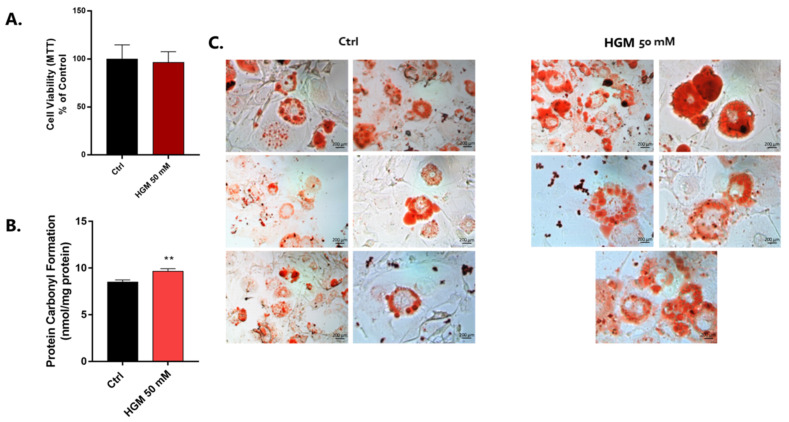
High-glucose medium affects adipocyte differentiation. The 50 mM high-glucose medium was used to expose pre-adipocyte cells for stimulation to differentiation. Firstly, cytotoxicity was measured by using an MTT assay (**A**). Thereafter, oxidative stress was measured by evaluating the protein carbonylation (nmol/mg protein) by performing a method using DNPH (2,4-dinitrophenylhydrazine derivatizing agent) (**B**). The data are plotted respecting mean ± SD (n = 3), and the significance was shown using Student’s *t*-test, ** *p = 0.0058*. Intracellular fat droplets of the pre-adipocytes were acquired using a light microscope (40× magnification) thereafter stained by using Oil Red O Staining (**C**). HGM: high-glucose medium.

To validate the proposed high-adipogenesis model, we first analyzed classical biomarkers of adipocyte phenotype in cells responding to high-glucose exposition, which did not affect their viability (Figure 2A). Oxidative stress markers and fat droplet staining were analyzed and correlated with inflammatory profile and adipogenesis. Reactive oxygen species and concomitant oxidative stress in adipocytes was better investigated by evaluating the protein carbonylation profile. Our data show that this parameter was significantly affected in cells responding to the high-glucose exposure, with higher values than the conventional condition (Ctrl) (Figure 2B). Images acquired by using light microscopy show that adipocytes responding to the high-glucose medium presented a higher number of bigger-sized intracellular fat droplets stained by Oil Red O dye (Figure 2C).

**Figure 3:**
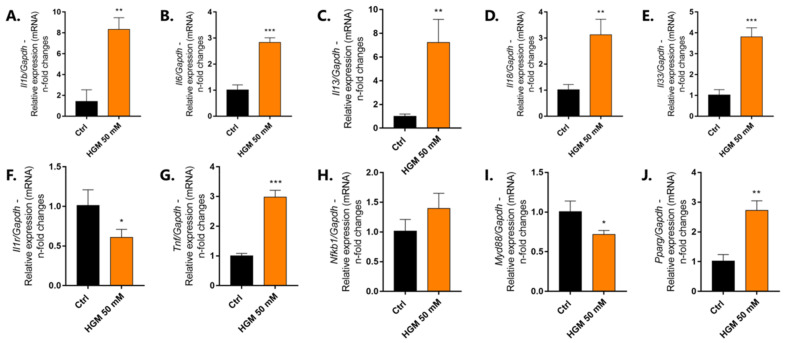
Adipogenesis model recapitulates the inflammation microenvironment and requires PPAR-γ. Pre-adipocytes were differentiated for 10 days using classical model when the cells were harvested and the biological samples forwarded to perform the qPCR technology. A significantly higher expression of IL-1β (**A**), IL-6 (**B**), IL-13 (**C**), IL-18 (**D**), IL-33 (**E**), TNF-α (**G**), and PPAR-γ (**J**) genes were observed in the adipocytes responding to HGM (50 mM). The graphs bring the n-fold change of the profile of gene expression normalized to the GAPDH gene (housekeeping gene). Significant differences were considered when * *p* < 0.05, and ** *p* < 0.01, *** *p* < 0.001. HGM: high glucose medium.

Adipocyte differentiation and inflammation signaling pathways are widely studied regarding obesity concerns. Herein, the gene expression was evaluated by investigating the behavior of the interleukins IL-1β, IL-6, IL-13, IL-18, and IL-33 genes. They were significantly higher in cells responding to the adipogenesis model where pre-adipocytes were exposed to the high-glucose medium (HGM) (Figure 3A–E), as well as considering TNF-α gene expression (Figure 3G). Importantly, the expression activity of the PPAR-γ gene was also investigated in this study. It was significantly higher in cells responding to HGM (Figure 3J), while both Myd88 and IL1 receptor genes expression were lower in the HGM group (Figure 3F,I), and NFkB remains unchanged (Figure 3H).

**Figure 4:**
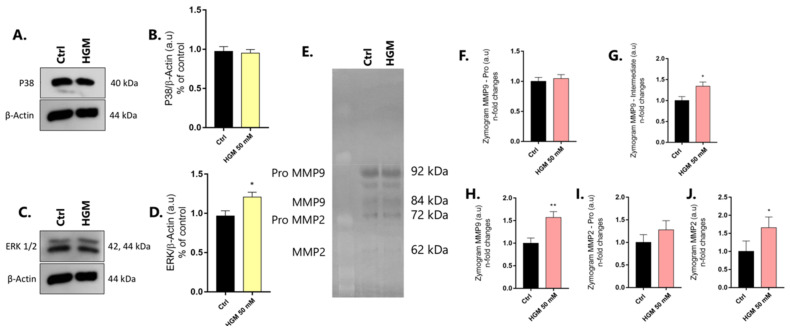
Adipocytes require MAPK and MMP activity. Adipocytes require MAPK-ERK (**A**,**B**), while the MAPK-P38 protein remains unchanged (**C**,**D**). β-Actin was used as the protein loading control. Additionally, higher activities of MMP9 (**F**–**H**) and MMP2 (**I**,**J**) were found in adipocytes responding to HGM. Data are plotted as means ± standard deviations (n = 3). Significant differences were considered when * *p* < 0.05, and ** *p* < 0.01. HGM: high glucose medium.

We also investigated the behavior of mitogen-activated protein kinase (MAPKs) genes to infer about cell survival signaling in differentiated adipocytes. Our data show that there is a higher profile of MAPK-ERK proteins in cells responding to HGM (Figure 4A,B), while the MAPK-P38 protein remains unchanged (Figure 4C,D). Finally, the perspective of extracellular matrix (ECM) remodeling was investigated by analyzing the activities of matrix metalloproteinases (MMPs) through gelatin-based proteolysis assay. Our data show that there is higher activity of both MMP2 and 9 in differentiated adipocytes (Figure 4E–J).

### 3.2. Angiogenesis-Related Genes Were Evaluated in ECs Responding to High Adipogenesis and Titanium

**Figure 5:**
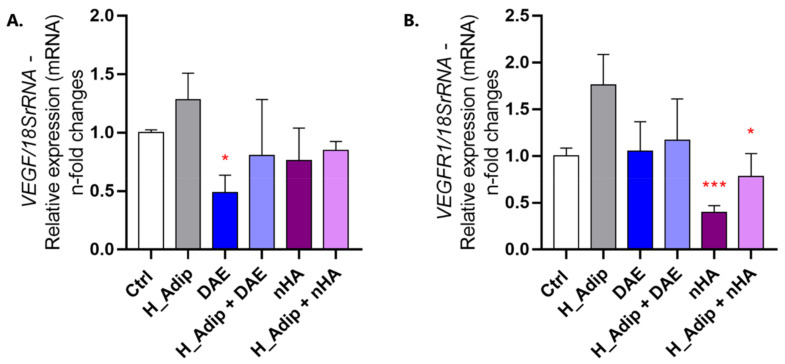
VEGF and VEGFR1 genes are modulated in response to titanium-based surfaces. Both genes are related to EC phenotype as well as to angiogenesis. Their response to H_Adip and the titanium-enriched medium seems to be relevant to the lower angiogenesis profile in adipogenesis. The VEGF gene presented a low expression profile in ECs responding to DAE, while there was no significance when considering the other groups (**A**). Additionally, the VEGFR1 gene presented a low profile of expression in ECs responding to nHA (**B**). The data show the n-fold changes in the profile of transcripts normalized to the 18 S gene (housekeeping gene). Differences were considered statistically significant when * *p* < 0.05, and *** *p* < 0.001, represented by red * when compared to the H_Adip group.

To analyze the behavior of ECs responding to high-adipogenesis and titanium-enriched mediums, we first evaluated angiogenesis-related genes and viability. The Ctrl group now refers to the adipocyte-conditioned medium subjected to normal conditions, while the H_Adip group refers to the adipocyte-conditioned medium chronically responding to high glucose concentrations (50 mM); furthermore, the DAE and nHA refer to the adipocyte-conditioned medium previously used to incubate titanium discs with respect to the difference on their surfaces: H_Adip + DAE and H_Adip + nHA. The VEGF gene remains unchanged when compared to Ctrl or when the cells were treated with either of the titanium-enriched mediums (Figure 5A). In this way, the VEGFr1 gene remains unchanged when compared to the Ctrl group, but significantly decreases in response to both nHA and H_Adip + nHA groups when compared to H_Adip (Figure 5B).

### 3.3. Proliferation and Survival-Related Genes in ECs

**Figure 6:**
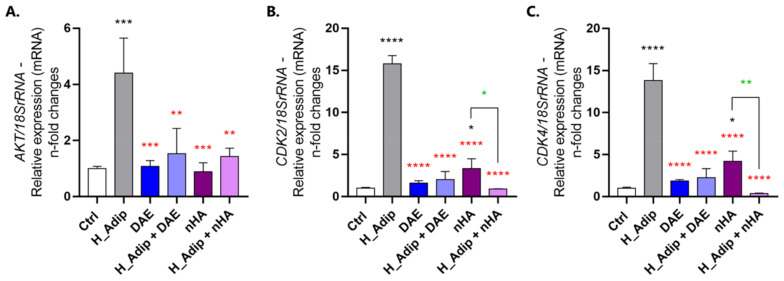
Both survival and cell proliferation progress were investigated in ECs. ECs require an increase in survival and cell cycle-related gene expression even more responding to high-adipogenesis condition, observing the behavior of AKT (**A**), CDK2 (**B**), and CDK4 (**C**). Differences were considered statistically when * *p* < 0.05, ** *p* < 0.01, *** *p* < 0.001, and **** *p* < 0.0001, represented by black * when compared to the Ctrl group, by red * when compared to the H_Adip group, and by green * when compared between the groups with nHA.

The protein kinase B (AKT), cyclin-dependent kinase 2 (CDK2), and CDK4 genes were significantly higher in ECs exposed to adipocyte-conditioned medium (H_Adip) (Figure 6A–C). Thereafter, our data show that there is a significant involvement of the AKT gene in the coupling of adipogenicity and titanium-enriched medium (Figure 6A). Thereafter, the CDK genes presented a very similar profile between DAE and nHA, with CDK2 being higher in nHA (Figure 6B), and CDK4 expressing a very similar profile (Figure 6C).

**Figure 7:**
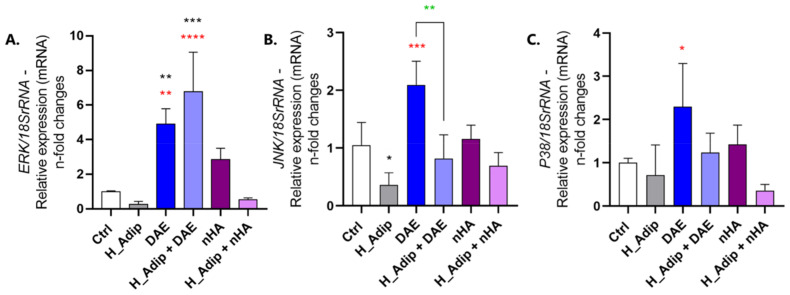
MAPK-related genes were modulated in EC responding to adipogenesis. qPCR shows different modulations of the MAPKs genes in ECs: the MAPK-ERK gene was higher in cells responding to the DAE group, and even higher in the H_Adip + DAE group. However, there was no difference in the H_Adip + nHA group when compared to Ctrl (**A**). The JNK was down-regulated in the H_Adip group when compared to Ctrl, and higher in response to DAE treatment when compared to H_Adip. Data are reported as means ± standard deviations (n = 3). Comparison by one-way ANOVA. Statistical differences were considered when * *p* < 0.05, ** *p* < 0.01, *** *p* < 0.001, and **** *p* < 0.0001, represented by black * when compared to the Ctrl group, by red * when compared to the H_Adip group, and by green * when compared between the groups with DAE.

MAPK genes were also investigated in ECs. Figure 7 shows there is a significant modulation in response to the coupling between high-adipogenicity and titanium-enriched medium. The titanium DAE-enriched medium increased the expression of the MAPK-ERK gene independently of normal or high-adipogenicity conditions (Figure 7A). ECs required MAPK-JNK gene expression when they were exposed to the DAE-enriched medium under normal adipogenesis conditions (DAE) when compared to H_Adip group, but without difference in the H_Adip + DAE group, while cells responding to nHA and H_Adip + nHA remain unchanged (Figure 7B). The MAPK-P38 gene showed involvement but without statistically significant changes when the groups were compared to the Ctrl group, showing an increase only in the DAE group when compared to H_Adip (Figure 7C).

**Figure 8:**
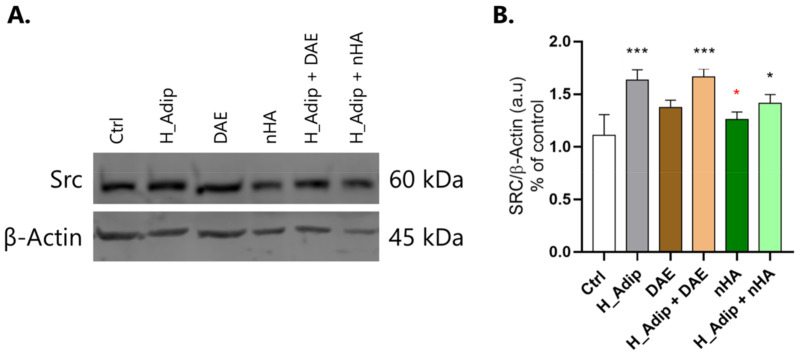
c-Src involvement. After exposing the ECs to different shear-stress treatments for 72 h, the samples were obtained to perform the Western blotting assay and evaluate SRC protein content (**A**,**B**). The high adipogenesis condition increased the content of SRC in ECs, significantly in the H_Adip + DAE group, and less significantly in the H_Adip + nHA group. β-Actin was considered the protein loading control. Differences were considered significant when * *p* < 0.05, and *** *p* < 0.001, represented by black * when compared to the Ctrl group, and by red * when compared to the H_Adip group.

As c-Src kinase is related to the survival mechanism governing the viability of eukaryotic cells, we decided to evaluate whether this protein was involved in response to the titanium-enriched medium under normal and high-adipogenesis conditions. Additionally, our data show that c-Src seems to be required in high-adipogenesis conditions regardless of the presence of the titanium DAE-enriched medium as it remained higher in the group H_Adip + DAE (Figure 8A,B). Moreover, in the titanium nHA-enriched medium, the cells under high adipogenesis showed a significant difference when compared with Ctrl (Figure 8).

### 3.4. Endothelial Cell Appears to Be Important in Inflammatory Gene Microenvironment

**Figure 9:**
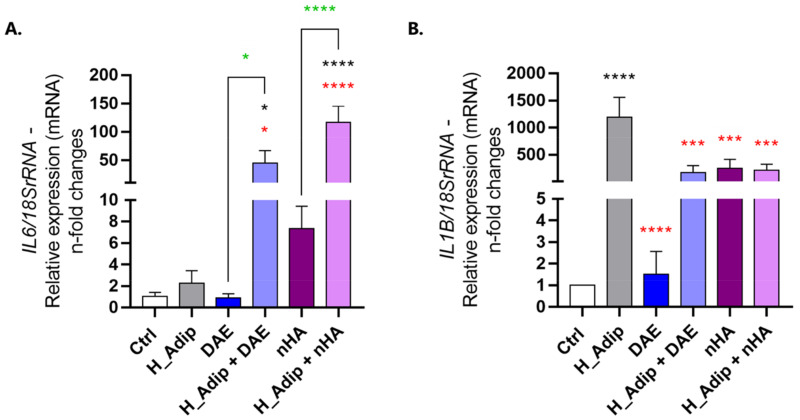
IL6 and IL1B gene expression changed in response to conditioned mediums. The samples were harvested as described earlier and the gene expression was measured using qPCR. To address the inflammatory effect of different conditions on ECs, we evaluated IL-6 (**A**) and IL-1β (**B**) genes. The 18SrRNA gene was considered the housekeeping gene and used to normalize the expression values. Data are reported in means ± standard deviations (n = 3). Differences were considered significant when * *p* < 0.05, *** *p* < 0.001, and **** *p* < 0.0001, represented by black * when compared to the Ctrl group, by red * when compared to the H_Adip group, and by green * when compared between the groups with DAE or between the groups with nHA. DAE = titanium with Dual Acid-Etching; nHA = titanium with nano-Hydroxyapatite-coated surface.

The interleukins IL-6 and IL1-β genes were evaluated using RT-qPCR technology. This revealed an important modulation in ECs responding to different treatments. The IL-6 gene was significantly higher when ECs were treated with titanium DAE and nHA under the high-adipogenesis condition (Figure 9A). The IL-1β gene was also significantly higher in the high-adipogenesis condition, a situation that seems to be controlled in the presence of the titanium-enriched medium, whether considering DAE or nHA (Figure 9B).

## 4. Discussion

The experimental model proposed in this study permits the evaluation of the effect of high adipogenesis in the ECs responding to a titanium-enriched medium. The adipocyte differentiation under high-glucose conditions has been used as a tool in vitro to understand obesity-related metabolic dysfunctions [32], once the glucose enhances lipid accumulation and adipogenesis [33]. Regarding the validation of this alternative model, the adipocyte exposed to high glucose concentrations in our model significantly modulated specific interleukin gene expression as well as the PPAR-γ gene activation. These genes are related to adipocyte differentiation [34]. Taken together, this validates our biological model for obtaining functional adipocytes. Importantly, the increase in protein carbonylation in obtained adipocytes can be correlated with the high oxidative stress expected in differentiated adipocytes. In general, our proposed adipogenesis model promotes an increase in the intracellular fat droplets during the pre-adipocyte differentiation concomitantly with the increase in the pro-inflammatory profile, as expected in adipocytes [32,35]. Additionally, MAPK and PPAR are also both involved with adipocyte metabolism [33], which corroborates with our findings. Additionally, we have also shown significant morphological changes in adipocytes and which can explain the higher activities of MMPs and is expected to modulate the ECM remodeling. Considering in vitro studies, this experimental model presents limitations, such as evaluating the crosstalk between cells of different origins, however, it can be overcome by the evolutionarily conserved structure and functions of proteins and genes over 80%.

Although some progress has been made on the way to understanding the etiology of systemic and metabolic dysfunctions such as diabetes and obesity, their relevance to bone-healing peri-implants, which might explain the higher failure of implants in obese patients [36], is barely understood. In fact, it has been hypothesized that angiogenesis is compromised in obese and diabetic individuals. Thus, we have applied an experimental model to better evaluate the impact on ECs responding to a titanium-enriched medium which plays crucial roles during the osseointegration mechanism of angiogenesis, such as interacting with osteoblasts [37,38]. There are important similarities between osseointegration and wound healing, a situation that is harmful in patients with compromised metabolism [39]. To advance with this proposal, we have investigated the effect of two titanium-modified surfaces. Firstly, genes related to the phenotype of endothelial cells were investigated. While VEGF seemed not to be affected, its receptor, VEGFR1, was higher in ECs responding to the titanium-enriched medium. This might be explained by a correlation with its ligand and suggests an autocrine loop in this condition. The increase in the intracellular signaling upon VEGFR1 activation requires the involvement of MAPK upstream, modulating cell survival and proliferative phenotype. This explains the capacity of those cells to involve p38 and CDKs [40]. Additionally, the VEGFR1-related intracellular cascade seems to require Src kinase in this context, and also might be correlated with ECM remodeling by regulating MMP activity.

Lastly, the gene expression of interleukins IL-6 and IL-1β was shown to be sensitive to the response to titanium in an adipogenesis-related metabolic condition once both DAE and nHA promoted their higher expression. It is important that the IL-related cascade also requires the activation of MAPKs and Src. An important aspect is that IL-1β effectively and rapidly induces human mesenchymal stem cells differentiation into osteoblasts [41]. This might be an important axis coupling angiogenesis and osteogenesis during the osseointegration mechanism, meaning nHA is able to improve the capacity of ECs to drive bone healing in obese patients.

## 5. Conclusions

Taking our data into consideration, it is possible to suggest that nHA-coated surface favor biological events related to angiogenesis and might be an alternative strategy in adipogenesis-related metabolic conditions where usually the percentage of dental implant failure is higher. Altogether, this study gathers valuable information on understanding the higher failure of dental implants in obese individuals.

## Figures and Tables

**Table 1:** Sequences of the primers and conditions of the quantitative polymerase chain reaction cycle.

Genes	Primers	5′-3′ Sequences	Reaction’s Conditions
H-AKT	Forward	CAG CGC GGC CCG AAG GAC	95 °C, 3 s; 55 °C, 8 s; 72 °C, 20 s
	Reverse	GAC GCT CAC GCG CTC CTC TC	
H-CDK2	Forward	CTT TGC TGA GAT GGT GAC TCG	95 °C, 3 s; 55 °C, 8 s; 72 °C, 20 s
	Reverse	GCC TCC CAG ATT CCT CAT GC	
H-CDK4	Forward	CTC TCT AGC TTG CGG CCT G	95 °C, 3 s; 55 °C, 8 s; 72 °C, 20 s
	Reverse	GCA GGG ATA CAT CTC GAG GC	
H-ERK	Forward	GCA GCG CCT CCC TTG CTA GA	95 °C, 3 s; 55 °C, 8 s; 72 °C, 20 s
	Reverse	AAC AGC CTC TGG CCC ACC CAT	
H-IL1B	Forward	GGA GAA TGA CCT GAG CAC CT	95 °C, 3 s; 55 °C, 8 s; 72 °C, 20 s
	Reverse	GGA GGT GGA GAG CTT TCA GT	
H-IL6	Forward	AGT CCT GAT CCA GTT CCT GC	95 °C, 3 s; 55 °C, 8 s; 72 °C, 20 s
	Reverse	CTA CAT TTG CCG AAG AGC CC	
H-JNK	Forward	AAA GGT GGT GTT TTG TTC CCA GGT	95 °C, 3 s; 55 °C, 8 s; 72 °C, 20 s
	Reverse	TGA TGA TGG ATG CTG AGA GCC ATT G	
H-P38	Forward	GAG AAC TGC GGT TAC TTA	95 °C, 3 s; 55 °C, 8 s; 72 °C, 20 s
	Reverse	ATG GGT CAC CAG ATA CAC AT	
H-VEGF	Forward	TGC AGA TTA TGC GGA TCA AAC C	95 °C, 3 s; 55 °C, 8 s; 72 °C, 20 s
	Reverse	TGC ATT CAC ATT TGT TGT GCT GTA G	
H-VEGFr1	Forward	CAG GCC CAG TTT CTG CCA TT	95 °C, 3 s; 55 °C, 8 s; 72 °C, 20 s
	Reverse	TTC CAG CTC AGC GTG GTC GTA	
M-Gapdh	Forward	AGG CCG GTG CTG AGT ATG TC	95 °C, 3 s; 55 °C, 8 s; 72 °C, 20 s
	Reverse	TGC CTG CTT CAC CAC CTT CT	
M-Il13	Forward	CAG TCC TGG CTC TTG CTT G	95 °C, 3 s; 55 °C, 8 s; 72 °C, 20 s
	Reverse	CCA GGT CCA CAC TCC ATA CC	
M-Il18	Forward	ACT TTG GCC GAC TTC ACT GT	95 °C, 3 s; 55 °C, 8 s; 72 °C, 20 s
	Reverse	GGG TTC ACT GGC ACT TTG AT	
M-Il1b	Forward	GAC CTT CCA GGA TGA GGA CA	95 °C, 3 s; 55 °C, 8 s; 72 °C, 20 s
	Reverse	AGC TCA TAT GGG TCC GAC AG	
M-Il1r	Forward	ACC CCC ATA TCA GCG GAG CG	95 °C, 3 s; 55 °C, 8 s; 72 °C, 20 s
	Reverse	TTG CTT CCC CCG GAA CGT AT	
M-Il33	Forward	CCT TCT CGC TGA TTT CCA AG	95 °C, 3 s; 55 °C, 8 s; 72 °C, 20 s
	Reverse	CCG TTA CGG ATA TGG TGG TC	
M-Il6	Forward	AGT TGC CTT CTT GGG ACT GA	95 °C, 3 s; 55 °C, 8 s; 72 °C, 20 s
	Reverse	CAG AAT TGC CAT TGC ACA AC	
M-Myd88	Forward	ATG GTG GTG GTT GTT TCT GAC GA	95 °C, 3 s; 55 °C, 8 s; 72 °C, 20 s
	Reverse	GCA AGG GTT GGT ATA GTC GCA TAT A	
M-Nfkb	Forward	CAC CTG TTC CAA AGA GCA CC	95 °C, 3 s; 55 °C, 8 s; 72 °C, 20 s
	Reverse	GGT TCA GGA GCT GCT GAA AC	
M-Pparg	Forward	TTT TCA AGG GTG CCA GTT TC	95 °C, 3 s; 55 °C, 8 s; 72 °C, 20 s
	Reverse	AAT CCT TGG CCC TCT GAG AT	
M-Tnf	Forward	CCA CAT CTC CCT CCA GAA AA	95 °C, 3 s; 55 °C, 8 s; 72 °C, 20 s
	Reverse	AGG GTC TGG GCC ATA GAA CT	

Note: H = Human; M = Mice.

## Data Availability

The data that support the findings of this study are available from the corresponding author upon reasonable request.
